# Subpopulation composition of monocytes and inflammation markers in schizophrenia

**DOI:** 10.1192/j.eurpsy.2023.2117

**Published:** 2023-07-19

**Authors:** S. A. Zozulya, Z. V. Sarmanova, I. N. Otman, I. V. Oleichik, T. P. Klyushnik

**Affiliations:** 1 Laboratory of Neuroimmunology; 2 Department of Endogenous Mental Disorders and Affective States, Mental Health Research Centre, Moscow, Russian Federation

## Abstract

**Introduction:**

Taking into account the role of the immune system in the pathogenesis of schizophrenia, it is important to study the peculiarities of innate immunity in the development of the disease. A special role in these processes belongs to monocytes, which play an integral role in the inflammatory reactions and perform regulatory and effector functions to other immunocytes.

**Objectives:**

To analyze the subpopulation composition of monocytes and other inflammatory markers in patients with schizophrenia

**Methods:**

The study included 36 women with schizophrenia (F20, ICD-10) (30±12 years) in the acute stage of the disease and 20 healthy donors. Flow cytometry was used to determine the relative number of monocyte subpopulations. The activity of leukocyte elastase (LE) and α1-proteinase inhibitor (α1-PI) in blood was determined by spectrophotometric method. The level of autoantibodies to S100b and CRP concentration were assessed by ELISA.

**Results:**

A decrease in “classical” monocyte subpopulation (p=0.02) was accompanied by an increase in cells of the proinflammatory phenotype (p=0.03) “Transitional” and “non-classical” subpopulations did not differ from controls. A negative correlation was found between the proportion of “classical” monocytes with “transitional” and “intermediate” cells (r=-0.66 and r=-0.54, p=0.01). All inflammatory and autoimmune blood markers in patients were significantly elevated compared to controls (p<0.05).

**Conclusions:**

The redistribution of the subpopulation composition of monocytes with an increase in “intermediate” subpopulation and an increase in other immune markers in the acute stage of schizophrenia serve as an additional link confirming the involvement of cellular immunity in the pathogenesis of the disease.

**Disclosure of Interest:**

None Declared

